# Deaths during the first year of the COVID-19 pandemic: insights from regional patterns in Germany and Poland

**DOI:** 10.1186/s12889-022-14909-9

**Published:** 2023-01-26

**Authors:** Michał Myck, Monika Oczkowska, Claudius Garten, Artur Król, Martina Brandt

**Affiliations:** 1 Centre for Economic Analysis (CenEA), ul. Cyfrowa 2, 71-441 Szczecin, Poland; 2 University of Greifswald, 17489 Greifswald, Germany; 3 Institute for the Study of Labor, 53113 Bonn, Germany; 4 TU Dortmund University, August-Schmidt-Straße 4, 44227 Dortmund, Germany

**Keywords:** COVID-19 pandemic, excess deaths, Spatial correlation, Public health policies

## Abstract

**Background:**

Given the nature of the spread of SARS-CoV-2, strong regional patterns in the fatal consequences of the COVID-19 pandemic related to local characteristics such as population and health care infrastructures were to be expected. In this paper we conduct a detailed examination of the spatial correlation of deaths in the first year of the pandemic in two neighbouring countries – Germany and Poland, which, among high income countries, seem particularly different in terms of the death toll associated with the COVID-19 pandemic. The analysis aims to yield evidence that spatial patterns of mortality can provide important clues as to the reasons behind significant differences in the consequences of the COVID-19 pandemic in these two countries.

**Methods:**

Based on official health and population statistics on the level of counties, we explore the spatial nature of mortality in 2020 in the two countries – which, as we show, reflects important contextual differences. We investigate three different measures of deaths: the officially recorded COVID-19 deaths, the total values of excessive deaths and the difference between the two. We link them to important pre-pandemic regional characteristics such as population, health care and economic conditions in multivariate spatial autoregressive models. From the point of view of pandemic related fatalities we stress the distinction between direct and indirect consequences of COVID-19, separating the latter further into two types, the spatial nature of which is likely to differ.

**Results:**

The COVID-19 pandemic led to much more excess deaths in Poland than in Germany. Detailed spatial analysis of deaths at the regional level shows a consistent pattern of deaths officially registered as related to COVID-19. For excess deaths, however, we find strong spatial correlation in Germany but little such evidence in Poland.

**Conclusions:**

In contrast to Germany, for Poland we do not observe the expected spatial pattern of total excess deaths and the excess deaths over and above the official COVID-19 deaths. This difference cannot be explained by pre-pandemic regional factors such as economic and population structures or by healthcare infrastructure. The findings point to the need for alternative explanations related to the Polish policy reaction to the pandemic and failures in the areas of healthcare and public health, which resulted in a massive loss of life.

**Supplementary Information:**

The online version contains supplementary material available at 10.1186/s12889-022-14909-9.

## Background

The COVID-19 pandemic took and still takes a huge economic, health and social toll across societies worldwide – with substantial variation over time and between continents, countries and regions. The regional variation of different incidences, conditions, measures and their outcomes is yet to be described, explored and analysed in order to learn lessons for the ongoing and future pandemics.

In this paper, we provide a piece to the puzzle by exploring the regional and spatial nature of deaths during the first year of the COVID-19 pandemic in Germany and Poland, two neighbouring countries which with regard to excess deaths in 2020 were placed at the opposing ends on a list of 29 high income countries [1]. Taking EUROSTAT death statistics in 2020, excess deaths rate in Poland, with reference to the 2016-2019 average, was 194 per 100,000 inhabitants, while the rate calculated for Germany was 62 [2, 3]. At the same time with a population 2.2 times greater compared to Poland in Germany the death toll directly linked to COVID-19 in 2020 amounted to 50,447 [4] – 1.7 times higher than the total number for Poland of 28,479 [5]. These large differences at the country level constitute the key motivation for the comparison of the two countries. We focus on the variation in the spatial patterns of deaths and show that the identified differences provide valuable insights regarding the reasons behind the aggregate numbers of fatalities.

Additionally several important characteristics of the two countries make the comparison particularly interesting. The pre-pandemic conditions in Poland and Germany with regard to the socio-demographic structure of the population as well as health-care and public health were substantially different. Moreover, within each country there was substantial regional heterogeneity in these conditions which may have made the risks of death due to SARS-CoV-2 much higher in some regions compared to others throughout the pandemic [6]. This high regional variation in risk factors, combined with massively different aggregate fatal consequences of the pandemic, constitutes a unique opportunity to explore the regional dimension with the aim of developing insights into reasons behind the dramatic differences in the overall number of deaths.

In the following, we explore the spatial nature of deaths in each country – which, as we show, reflects important contextual variation. We focus on the spatial patterns of mortality and examine the relationship of excessive deaths in the first year of the pandemic with pre-pandemic local characteristics on the level of *Kreise* in Germany and *powiaty* in Poland (hereafter also referred to as “counties”). We analyse three different measures of deaths: the officially recorded COVID-19 deaths, the total values of excessive deaths and the difference between the two, and link them to important regional characteristics such as population, health care and economic conditions in multivariate spatial autoregressive models.

The paper is structured as follows. We first provide some background to the spatial nature of the COVID-19 pandemic. This is followed by a description of the methods applied and data used in the paper leading to the analysis of the spatial distribution of COVID-19 and excess deaths jointly with the spatial nature of potentially important regional influences. Results showing the findings based on multivariate spatial models are followed by a discussion and conclusions.

### The spatial aspect of COVID-19 pandemic risks: state of research

As SARS-CoV-2 started spreading in the late 2019 and early 2020, it became clear that individuals particularly at risk of severe consequences of COVID-19 are older people and those with long-term cardiovascular [7, 8] and pulmonary conditions [9] as well as those with generally weakened immune responses [10], in particular those suffering from cancer [11]. The ability of the virus to transmit through air via droplets and in aerosols makes infections more probable in contexts in which many people are gathered in small unventilated spaces. This applies not only to public transport and the workplace [12], but also to living contexts of overcrowded housing [13], though linking many specific factors for the spread of COVID-19, like intergenerational relationships and co-residence, has proved to be a challenge [14].

The nature of contagious diseases which cause pandemics is inseparable from the spatial context, and the spatial dimension is crucial to understand the spread of infections, its consequences, and the effectiveness of measures which limit it [15–22]. Since the early start of the COVID-19 pandemic spatial analyses have also been part of the research on SARS-CoV-2. This included the regional spread of the virus [21, 23–27], geographical accessibility to healthcare infrastructure [24, 28], effectiveness of regional policies for non-pharmaceutical interventions [29–31], as well as the importance of environmental and socioeconomic factors and their role in the spread of the virus [22, 30, 32–35]. According to findings from a regional level analysis in five European countries, limiting virus transmission around large transportation hubs was especially important at the early stages of the pandemic [25]. Analysis at the regional level on Dutch data showed that while hospitalization and excess mortality were not related during the first wave in 2020, excess mortality was lower in regions that had continued a stricter strategy of containment [36]. For Italy, greater availability of intensive care beds correlated with lower COVID-19 mortality during the first wave in 2020 [16]. Another regional study on Italy emphasized the remarkably uneven distribution of excess deaths in the country, with 3 out of 107 provinces accounting for over 30% of these deaths [37].

Several early findings on Poland and Germany have also taken a regional focus. For example, in Poland in the early months of the pandemic incidence and COVID-19 mortality was highest in the (post-)industrial region of Silesia. This was partly due to mass testing of miners at the time [38], which in turn was a reaction to identified risks of contagion in mines. The spread of COVID-19 in Silesia was reflected in the number of hospital admissions and deaths. Those more likely to die were older men, and individuals with chronic cardiovascular or respiratory diseases [39]. Moreover, Polish regions with higher air pollution experienced higher incidence of COVID-19 and related deaths [40]. In Germany 12 of 16 regions experienced increased mortality for some weeks during the first wave of 2020, but not for the period between January and June [41]. Life expectancy in 2020 decreased especially in the eastern part of Germany among men over the age of 65 [42]. A higher number of reported COVID-19 cases and deaths was positively related to the numbers of personnel in nursing homes and average age of population, while the share of people aged 75 and older was negatively related to cases and deaths during the first wave [43]. Age corrected excess mortality in relation to years 2016 to 2019 was low for 2020 in total, but this was because mortality was below the expected during summer months and increased in winter during the second wave of the pandemic [44]. It was during the second wave that more dense regions, those with lower average income, higher employment rates and higher percentage of employed in the production industry had higher incidences [45].

Taken together, apart from a “random” component of the virus’s spread, population age structure and healthcare infrastructure linked to economic circumstances are likely to play a decisive role in determining the fatal consequences of the pandemic in a region. Up to now, however, there is no analysis linking all these factors in a detailed spatial analysis of mortality during the pandemic for Germany and/or Poland.

## Methods

The nature of COVID-19 and the high degree of regional variation between and within the two countries along some crucial dimensions make Germany and Poland an interesting international case for comparison and analysis of the pandemic’s consequences. We focus on the 401 *Kreise* as regional units in Germany and the 380 *powiaty* as units in Poland.^1^ The analysis is conducted at the county level with a primary focus on the pattern of regional spatial correlation of deaths in the first year of the pandemic. We examine these patterns separately for each country, and in each case for three outcome variables: COVID-19 deaths, total excess deaths and the difference between the two.

### Uncovering the spatial nature of excess deaths in Germany and Poland

The key objective of the estimations presented in this paper is to identify the degree of spatial correlation in the examined measures [46]. Spatial similarities among regions are of course present along many dimensions, but are particularly important in discussing such phenomena as pollution or pandemics where the examined outcomes spread across physical space affecting close-by regions to a greater extent compared to more distant ones. Such spatial correlation results in clustering among regions, with certain areas becoming ‘hot-’ or ‘cold-spots’ with higher or lower than average values of certain characteristics. To test for spatial dependence we first run simple OLS models and examine the spatial correlation of the residuals. This is then followed by estimations using spatial autoregressive models to account for the spatial correlation of the residuals (spatial error models) and of the dependent variables (spatial lag models).

The pattern of spatial correlation is examined using different spatial weight matrices (SWM) to test spatial correlation among the first and second order nearest neighbours, using fixed distance as well as inverse-distance weight matrices based on the distance between the centroids of counties with a range of truncation thresholds. For each of these matrices we estimate the significance of global Moran’s I test [47] with results reported in Table S1 in the Additional file 2. Given the nature of COVID-19 and outcomes of the tested approaches, as our preferred model we chose the specification with the row normalized inverse distance weight matrix truncated at the 70 km threshold, the shortest threshold which allows us to account for all counties in both countries. Overall, the significance levels of the Moran’s I statistic for the spatial correlation of the three variables of interest (without controls) using the selected matrix turn out to be very similar compared to those estimated using other approaches (see Table S1 in the Additional file 2).

### Direct and indirect relationship of deaths to the COVID-19 pandemic

We examine three outcomes related to mortality in 2020: the official number of COVID-19 deaths, the number of excess deaths, and the difference between the two. Both in Germany and Poland for registration and reporting of COVID-19 related mortality, the official approach has been to apply a broad definition of COVID-19 deaths, which included all cases with identified SARS-CoV-2 infection, regardless whether the person died from COVID-19 or with it - for another main reason.^2^ For the purpose of this analysis, in both countries we aggregated daily statistics of COVID-19 deaths in 2020 for each county. Excess deaths are measured as the difference in the number of total deaths in year 2020 and the average number of deaths between 2015 and 2019 (this definition is similar to that used to calculate excess mortality by the EUROSTAT; 2). The difference is calculated as a simple subtraction of COVID-19 deaths from total excess deaths. All three outcomes are measured at the county level and are calculated per 1000 individuals of the regional population in 2019 (for details of data sources see the Data Statement section).

For the purpose of this paper, we define the relationship of deaths to the COVID-19 pandemic in the following fashion. By ‘direct’ fatal consequences of the pandemic we mean all deaths that were the immediate consequences of being infected with SARS-CoV-2, i.e. all those who were infected with the virus and died before recovering from COVID-19. This group includes all those who were officially identified in the COVID-19 statistics, as well as those who died as a direct consequence of the infection but were not registered (in our data the latter group would be part of the ‘excess deaths’ category but not of the ‘COVID-19 deaths’ category). An important distinction that we make here concerns deaths which have been indirectly caused by the COVID-19 pandemic. In particular we distinguish indirect consequences of ‘type 1’ as those premature deaths which did not follow an infection, but which occurred due to the spread of COVID-19. This category covers, for example, deaths resulting from lack of hospital beds because of the number of local COVID-19 patients, limitations in medical procedures in heavily affected regions, or lack of medical personnel resulting from SARS-CoV-2 infections and locally imposed quarantines. Indirect consequences of ‘type 2’ in turn are deaths which would not have occurred had the pandemic not happened, but which do not necessarily relate to the actual spread of the virus. These additional deaths would include fatalities resulting, for example, from externally or self-imposed restrictions such as access to primary health care, reduced contact with other people, diminished family support, mental health problems due to isolation, etc. The distinction between type 1 and type 2 indirect deaths is crucial from the point of view the spatial dimension of the pandemic. While type 1 indirect deaths could be expected to follow the spatial distribution of the virus, such patterns would not necessarily be observed for type 2 indirect deaths.

## Results

### Regional variation in deaths in 2020

**Fig. 1:**
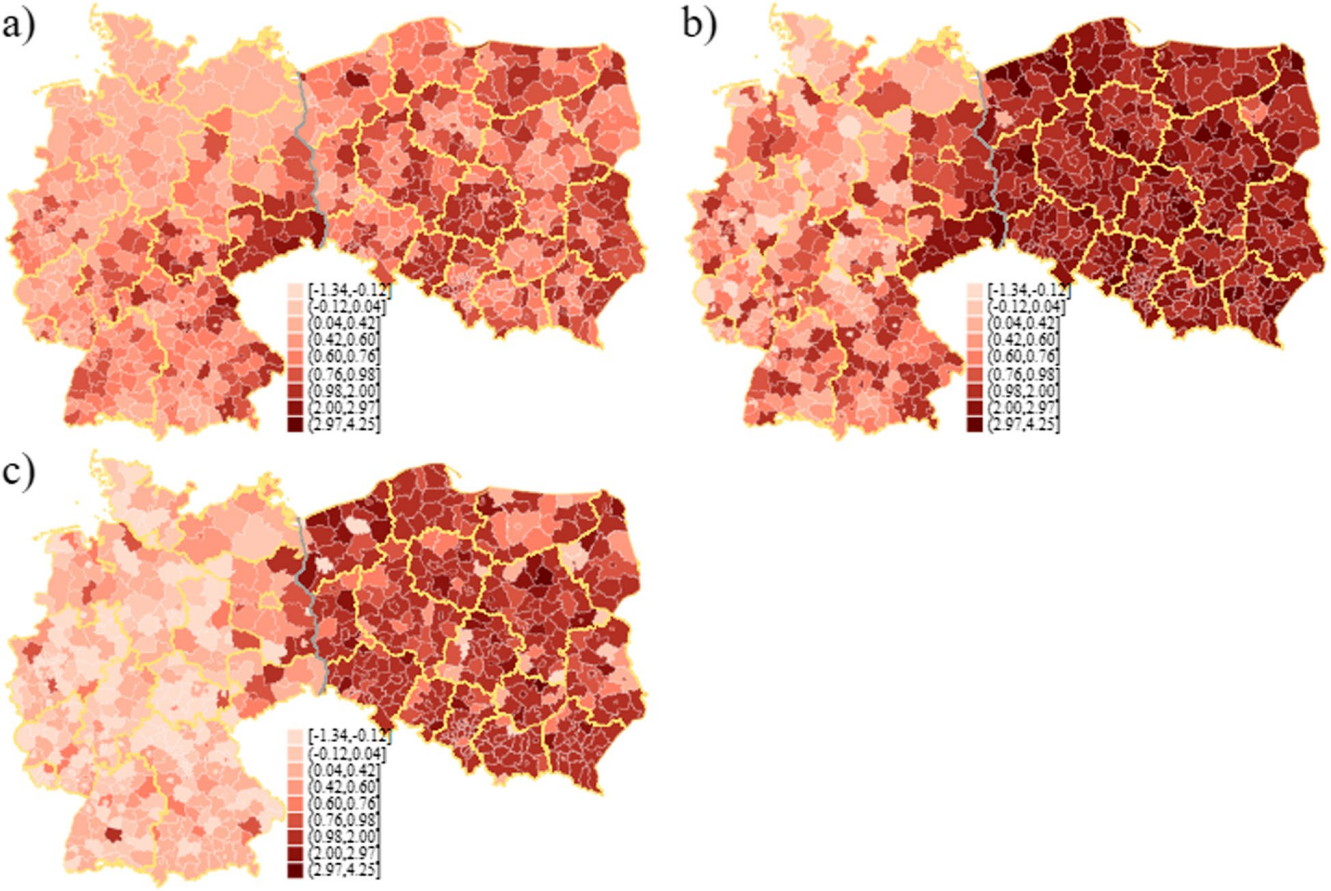
Regional variation of death incidence in 2020: Germany and Poland. **a** COVID-19 deaths per 1000 population. **b** Excess mortality per 1000 population. **c** Difference in excess and COVID-19 deaths per 1000 population. *Source:* own compilation based on (i) Germany: geodata: Federal Agency for Cartography and Geodesy (BKG); statistics: Robert Koch Institute (RKI), Federal and state statistical offices; (ii) Poland: geodata: National Register of Boundaries (PRG); statistics: regional and local Sanitary Inspectorates, Ministry of Health, Local Data Bank (BDL). *Notes:* The panels share a common legend based on the quintile distribution of COVID deaths, with two additional categories added at the top and bottom of the scale. County borders in white, regional borders in yellow, country border in grey. For names of specific regions (*Länder* and voivodeships) see Fig. S1 in the Additional file 1

The regional distribution of COVID-19 related and excess deaths is presented in Fig. 1, with a common colour legend across the three definitions to facilitate the comparison of the regional variation both within and between the measures. Summary statistics are given also in Table 1. As we can see from Fig. 1, deaths officially registered as caused by COVID-19, excess mortality and differences between those two are generally much higher in Poland compared to Germany. On average (see Table 1) 0.63 persons per 1000 inhabitants died because of COVID-19 in Germany, while 0.86 persons per 1000 died in the average Polish *powiat*, although it is worth noting that regional variation in COVID-19 deaths is higher in Germany. Compared to COVID-19 deaths, excess mortality per 1000 inhabitants was only slightly higher in German regions (0.64), while it was much higher in Poland (2.19). Accordingly, the average difference between total excess deaths and COVID-19 deaths is close to 0 in Germany, and is as high as 1.33 in Poland.

**Table 1:** Pre-pandemic regional socio-economic indicators in 2019 and mortality indicators in 2020 on county level: Germany and Poland

	Germany (*Kreise* level)				Poland (*powiat* level)			
	Mean	SD	Min.	Max.	Mean	SD	Min.	Max.
*Pre-pandemic regional indicators in 2019:*								
Population structure by age group:								
- aged 50-69 (%)	29.79	2.91	21.43	37.18	26.14	1.61	20.99	30.07
- aged 70-84 (%)	13.44	1.76	9.29	20.03	9.29	1.32	5.95	14.86
- aged 85+ (%)	2.97	0.42	1.91	4.25	2.02	0.45	0.89	4.58
Population density (person/sqkm)	537.02	709.71	35.61	4777.04	368.84	655.15	19.02	3812.08
Number of hospital beds (per 1000 population)*	6.35	3.89	0	29.59	4.25	2.85	0	16.38
Employed in agriculture (%)	1.92	1.63	0	8.46	28.26	19.71	0.34	77.72
*Mortality indicators in 2020:*								
COVID-19 deaths per 1000 population	0.63	0.39	0.05	2.96	0.86	0.31	0.23	2.53
Excess mortality per 1000 population	0.64	0.58	−0.84	3.05	2.19	0.59	0.31	4.25
Difference in excess and COVID-19 deaths per 1000 population	0.00	0.44	−1.34	1.24	1.33	0.59	−1.26	3.11
Number of counties	401				380			

*Source:* see Figs. 1 and 2

*Notes:* * statistics from year 2016. Values presented in the table are arithmetic means (and their standard deviations) of local data. For some variables, such as population density, the harmonic mean is a more appropriate representation of the average – harmonic mean of population density for Germany is 172.54, while for Poland it is 84.53

Looking at the spatial distribution of 2020 deaths, in Poland regions with the highest number of deaths officially registered as caused by COVID-19 are concentrated in several voivodeships, for example the central łódzkie and south-eastern lubelskie voivodeships (for location of specific counties see Fig. S1 in the Additional file 1). In Germany, COVID-19 deaths seem to be concentrated heavily in the east and the south, foremost in Sachsen and Bayern. The difference in average excessive deaths, reported in Table 1, is reflected in a striking spatial pattern in Fig. 1b. Importantly, high excessive deaths in Poland can be noted also in regions where the number of COVID-19 deaths were lower. This characteristic is reflected in the spatial distribution of the difference between total excessive deaths and COVID-19 deaths in Fig. 1c. Excess mortality was predominantly high in German regions with a high number of COVID-19 deaths, but also regions close by show higher numbers of excess mortality and thus higher differences between excessive deaths and COVID-19 deaths. This is mostly the case in regions with high COVID-19 deaths or close to them (including Bayern and Sachsen, but also further north in Brandenburg).

A natural hypothesis with regard to the spatial nature of excess deaths in the first year of the pandemic is that the pattern of these deaths should reflect the nature of contagion. This should certainly apply to the deaths which were ‘direct’ consequences of the pandemic (whether officially recorded as COVID-19 deaths or not). However, the observed regional pattern of deaths, which were type 1 indirect consequences of COVID-19, should also reflect the spread of the virus: healthcare limitations, hospital beds shortages, etc., can be expected to be felt more strongly in regions with higher incidence of COVID-19. This suggests that if the spread of SARS-CoV-2 was responsible for higher than expected number of deaths, we should observe significant spatial patterns for the officially documented COVID-19 deaths and for the excess deaths not recorded in the official COVID-19 statistics, and hence, in consequence, also for the total number of excess deaths.

Such hot- and cold-spots are visible in the spatial pattern of deaths in 2020 (Fig. 1). In the latter case, clustering of counties with high numbers of COVID-19 deaths (Fig. 1a) is very distinct in the south-eastern part of Germany (Sachsen) and in central and eastern Poland (especially łódzkie and lubelskie voivodeships). Spatial clusters are also evident for excess mortality in Germany (especially in Sachsen and Brandenburg), while they are less obvious in Poland both for excess deaths and the difference between excess and COVID-19 deaths.

**Fig. 2:**
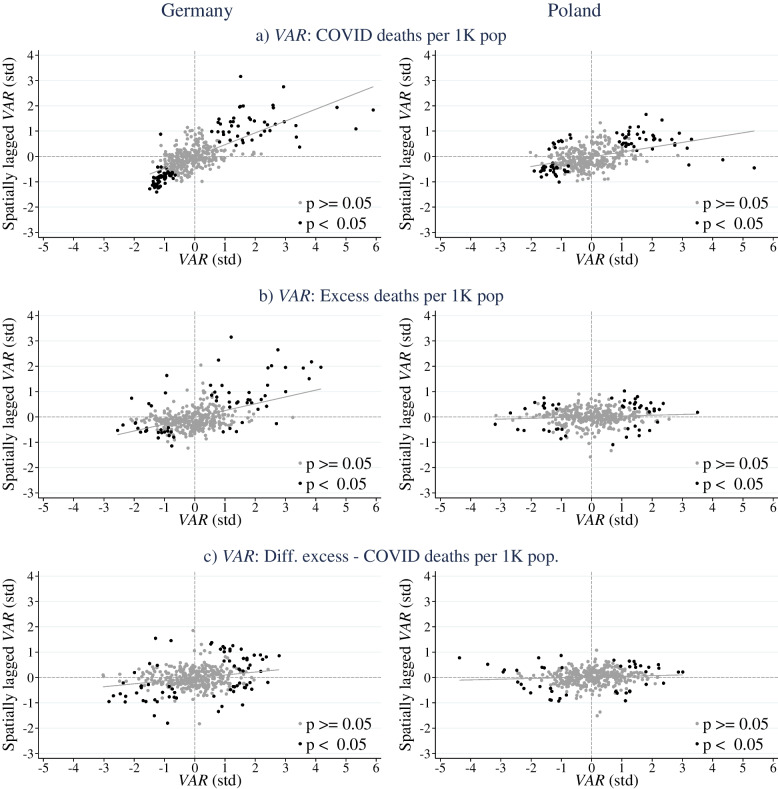
Local indices of spatial autocorrelation: Moran scatter plots of death measures. *Source:* own calculations based on county level data as described in *Source* notes for Figs. 1 and 3

Figure 2 is derived from local indices of spatial autocorrelation (calculated for each county). If the value of a local index for a given sub-area is higher (lower) compared to the value of the overall global index of spatial autocorrelation, then spatial autocorrelation is positive (negative). Spatial autocorrelation, here indicated by the slope of the regression line in the Moran scatterplots, is much higher in Germany than in Poland for all three death measures. In Poland, the slope of the regression line fitted into these scatterplots is statistically significant only for the COVID-19 deaths, and is essentially flat for total excess deaths and for the difference between total and COVID-19 deaths. Thus, while the pattern of spatial correlation in Germany for all three examined measures corresponds to the expected nature of deaths caused by the spread of SARS-CoV-2, this cannot be said about the pattern observed in Poland. Surprisingly, even though the regional numbers of total excess deaths in Poland are far higher, they do not reflect the expected spatial pattern. This suggests that a sufficiently high proportion of those deaths were not related to the spread of COVID-19, but rather represent indirect consequences of type 2 (according to the definition described in Section 2.2), which results in ‘blurring’ of the spatial pattern of deaths.

### Pre-pandemic regional variation in population and infrastructures

As shown in earlier research indicated above, counties in Germany and Poland differ significantly along a number of dimensions, which might be relevant for both the spread of the pandemic and the risk of death resulting from COVID-19. In both countries there are substantial regional differences in population density and age structure, the pattern of economic activity and variation in healthcare facilities, which may contribute to the different spatial patterns shown above.

**Fig. 3:**
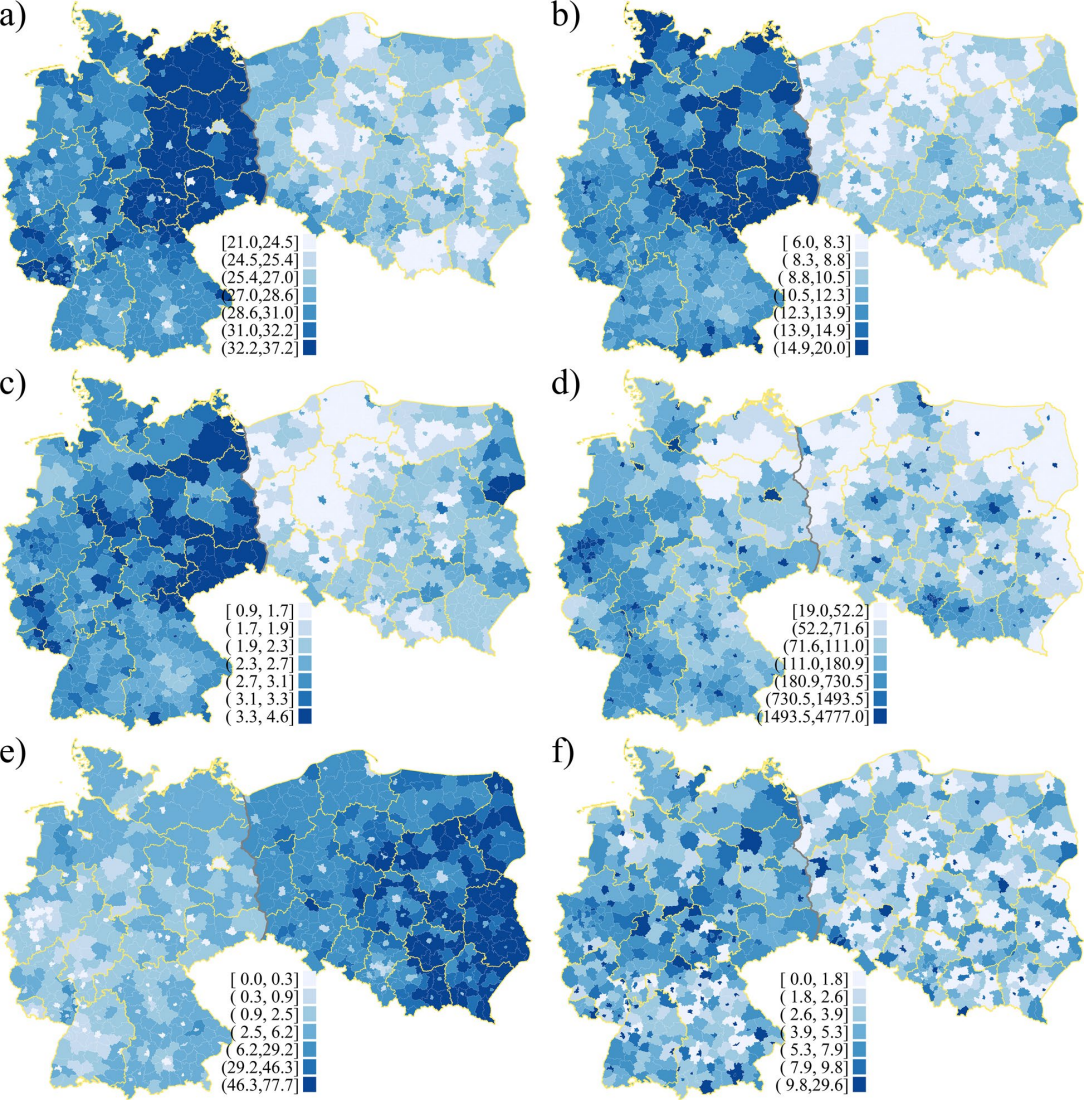
Pre-pandemic regional variation of socio-economic indicators in 2019: Germany and Poland. **a** Proportion of population aged 50-69. **b** Proportion of population aged 70-84. **c** Proportion of population aged 85+. **d** Population density (person/sqkm). **e** Proportion of employed in agriculture. **f** Number of hospital beds per 1000 population*. *Source:* own compilation based on (i) Germany: geodata: Federal Agency for Cartography and Geodesy (BKG); statistics: Federal and state statistical offices; (ii) Poland: geodata: National Register of Boundaries (PRG); statistics: Central Statistical Office (GUS). *Note:* * - statistics from year 2016. Two top and bottom categories in the legend cover 10% of observations each, the rest of categories cover 20% of observations each. County borders in white, regional borders in yellow, country border in grey. For names of specific regions (*Länder* and voivodeships) see Fig. S1 in the Additional file 1

Figure 3 shows spatial distributions of such socio-demographic indicators in years prior to the COVID-19 pandemic. Each of the maps in Fig. 3 combines the counties of Germany and Poland and the colour shading in each case reflects a common legend for both countries. The legend classes on each map have been set to reflect equal proportions of regions within each class. This approach makes both the between- and the within-country variations distinguishable, but naturally requires caution in interpreting the differences in values.^3^ As we can see in Fig. 3a, b and c, the age structure varies significantly between the two countries as well as within them. For each of the considered older age groups (respectively: 50-69, 70-84, 85+) the shares are clearly substantially higher in Germany compared to Poland. This reflects the differences in average values between the countries presented in Table 1. For example, the average proportion of the 50-69 group in Poland is 26.1% and in Germany it is 29.8%, while if we take the ‘oldest old’, those aged 85+, the average proportions are 2.0 and 3.0%, respectively. Overall, slightly higher shares of older people live in eastern Poland, and there are very clear differences between the German *Kreise* that formerly belonged to the German Democratic Republic (GDR) and the western part of Germany. The urban counties can be clearly distinguished in Fig. 3d, which shows the spatial distribution of population density (number of persons per square kilometre). One can note higher density levels in *powiaty* surroundings large cities in central and southern Poland, whereas in Germany population density is especially high in urbanized western and southwestern parts of the country. The proportion of people employed in agriculture (Fig. 3e) is significantly higher in Poland. The average proportion of workers employed in agriculture in Poland is 28.3%, while it is as low as 1.9% in Germany (Table 1). Employment in agriculture is more heavily concentrated in eastern Poland, while in Germany the share of agriculture employment is spread relatively evenly with slightly higher proportion in northern and southeast regions. In Fig. 3f we show the distribution of general healthcare infrastructure measured with the number of hospital beds per 1000 inhabitants. Naturally, the distribution reflects the urban/rural divide, with higher concentration of infrastructure in cities and counties with higher population density. The spatial pattern however, reflects another general difference between Poland and Germany, namely significantly better healthcare infrastructure, with the average numbers of hospital beds equal to 4.3 and 6.4 respectively (Table 1), a difference of nearly 50%. The scale of this discrepancy, while astounding, seems to be a good reflection of the differences in the overall quality of healthcare between the two countries. For example, while Germany is ranked 12th in the Euro Health Consumer Index [51], Poland comes as a distant 32nd country among the 35 for which this multidimensional healthcare quality index is computed.^4^

### Linking regional factors and the spatial dimension of deaths

In this section we examine results of multivariate spatial models to relate the adopted definitions of deaths with pre-pandemic regional factors, the socio-economic conditions introduced above. Results of the OLS and SAR estimations including diagnostic tests for spatial correlation using our preferred SWM are presented in Tables 2 (for Germany) and 3 (for Poland). The key results concerning spatial correlation are the following. As we can see in Table 2, in Germany all three outcomes show the expected statistically significant spatial pattern of correlation of the dependent variable. Additionally, the same is true for the spatial distribution of the residual. In the Polish case, the analysis also confirms statistically significant spatial correlation in the case of COVID-19 deaths (Table 3). However, for total excess deaths and for the difference between total excess and COVID-19 deaths, there is no significant spatial lag dependence. Similarly, in both cases the Lagrange Multiplier (LM) test suggests that there is no evidence for spatial correlation of the residuals, which is reflected also in Moran’s I statistics.

**Table 2:** Regression results – OLS compared with spatial autoregressive model, Germany

	COVID-19 deaths						Excess deaths						Difference: excess minus COVID-19 deaths					
	OLS		Spatial error model		Spatial lag model		OLS		Spatial error model		Spatial lag model		OLS		Spatial error model		Spatial lag model	
	coeff	SE	coeff	SE	coeff	SE	coeff	SE	coeff	SE	coeff	SE	coeff	SE	coeff	SE	coeff	SE
Pop. aged 50-69 (%)	0.012	(0.012)	0.024**	(0.011)	0.008	(0.009)	− 0.022	(0.018)	− 0.009	(0.019)	− 0.010	(0.016)	− 0.034**	(0.014)	− 0.034**	(0.015)	− 0.026*	(0.014)
Pop. aged 70-84 (%)	−0.017	(0.021)	−0.004	(0.021)	0.001	(0.015)	0.030	(0.031)	0.049	(0.036)	0.025	(0.027)	0.047**	(0.023)	0.050*	(0.027)	0.037	(0.022)
Pop aged 85+ (%)	0.302***	(0.086)	0.207**	(0.081)	0.150**	(0.064)	0.300**	(0.126)	0.141	(0.137)	0.172	(0.111)	−0.002	(0.095)	−0.044	(0.105)	−0.008	(0.092)
Employed in agricul. (%)	−0.030*	(0.016)	−0.021	(0.013)	−0.014	(0.012)	0.014	(0.023)	0.015	(0.023)	0.008	(0.020)	0.045**	(0.018)	0.039**	(0.018)	0.035**	(0.017)
Hospital beds (per 1 K)	−0.005	(0.006)	− 0.002	(0.004)	− 0.004	(0.004)	− 0.035***	(0.009)	− 0.028***	(0.008)	− 0.034***	(0.008)	− 0.030***	(0.007)	− 0.027***	(0.007)	− 0.030***	(0.006)
Pop. density (p/1000 sqm)	0.020	(0.039)	0.060*	(0.033)	0.029	(0.029)	−0.052	(0.057)	−0.055	(0.057)	−0.047	(0.050)	−0.072*	(0.043)	−0.100**	(0.046)	−0.073*	(0.042)
Constant	−0.321	(0.293)	−0.626**	(0.291)	−0.526**	(0.216)	0.217	(0.430)	0.029	(0.481)	−0.057	(0.378)	0.538*	(0.326)	0.617*	(0.364)	0.477	(0.315)
[depvar]					0.803***	(0.042)					0.619***	(0.061)					0.326***	(0.079)
e.[depvar]			0.821***	(0.040)					0.643***	(0.060)					0.358***	(0.083)		
var(e.[depvar])			0.075	(0.005)	0.077	(0.006)			0.233	(0.017)	0.236	(0.017)			0.163	(0.012)	0.164	(0.012)
*Diagnostics:*																		
*Measures of fit:*																		
AIC	364.044		161.350		168.395		670.503		593.035		596.511		447.569		434.763		435.959	
BIC	392.002		197.296		204.341		698.460		628.981		632.457		475.527		470.708		471.905	
*Tests for spatial error dependence:*																		
Lagrange multiplier stat.	417.418						148.799						25.007					
Lagrange multiplier *p*-value	0.000						0.000						0.000					
Moran’s I *z*-value	21.534						12.978						5.498					
Moran’s I *p*-value	0.000						0.000						0.000					
*Tests for spatial lag dependence:*																		
Lagrange multiplier stat.	382.926						138.228						23.996					
Lagrange multiplier *p*-value	0.000						0.000						0.000					
*Wald test of spatial terms:*																		
chi2			414.614		373.045				113.218		104.213				18.728		16.862	
Prob > chi2			0.000		0.000				0.000		0.000				0.000		0.000	

*Source:* own calculations based on county level data as described in *Source* notes for Figs. 1 and 3

*Notes:* Number of observations: 401. *p < 0.1, **p < 0.05, ***p < 0.01. Spatial error model – with spatially lagged errors, maximum likelihood estimator. Spatial lag model – with spatially lagged dependent variable, maximum likelihood estimator. The estimated variance inflation factor for conditioning variables varies between 1.55 and 3.77

**Table 3:** Regression results – OLS compared with spatial autoregressive model, Poland

	COVID-19 deaths						Excess deaths						Difference: excess minus COVID-19 deaths					
	OLS		Spatial error model		Spatial lag model		OLS		Spatial error model		Spatial lag model		OLS		Spatial error model		Spatial lag model	
	coeff	SE	coeff	SE	coeff	SE	coeff	SE	coeff	SE	coeff	SE	coeff	SE	coeff	SE	coeff	SE
Pop. aged 50-69 (%)	0.021*	(0.012)	0.026*	(0.014)	0.016	(0.011)	0.012	(0.024)	0.020	(0.026)	0.013	(0.024)	−0.009	(0.025)	− 0.003	(0.026)	− 0.007	(0.024)
Pop. aged 70-84 (%)	0.062***	(0.022)	0.049**	(0.024)	0.052**	(0.021)	0.057	(0.044)	0.041	(0.047)	0.050	(0.044)	− 0.005	(0.045)	− 0.018	(0.047)	− 0.009	(0.044)
Pop aged 85+ (%)	−0.110*	(0.060)	−0.064	(0.064)	−0.080	(0.056)	0.022	(0.118)	0.055	(0.125)	0.033	(0.117)	0.132	(0.120)	0.152	(0.125)	0.135	(0.119)
Employed in agricul. (%)	0.004***	(0.001)	0.002*	(0.001)	0.002**	(0.001)	−0.002	(0.002)	−0.003	(0.003)	−0.002	(0.002)	−0.006**	(0.002)	−0.007**	(0.003)	−0.006**	(0.002)
Hospital beds (per 1 K)	0.021***	(0.007)	0.015**	(0.007)	0.018***	(0.006)	0.025*	(0.014)	0.022	(0.014)	0.025*	(0.013)	0.003	(0.014)	0.003	(0.014)	0.004	(0.014)
Pop. density (p/1000 sqm)	−0.063*	(0.036)	−0.057	(0.036)	−0.061*	(0.034)	−0.098	(0.072)	−0.095	(0.073)	−0.095	(0.071)	−0.035	(0.073)	−0.035	(0.074)	−0.033	(0.072)
Constant	−0.234	(0.275)	−0.258	(0.318)	−0.425	(0.260)	1.282**	(0.545)	1.207**	(0.581)	1.012*	(0.580)	1.517***	(0.553)	1.439**	(0.590)	1.291**	(0.573)
[depvar]					0.489***	(0.080)					0.137	(0.108)					0.142	(0.109)
e.[depvar]			0.523***	(0.085)					0.172	(0.118)					0.181	(0.111)		
var(e.[depvar])			0.076	(0.006)	0.076	(0.006)			0.330	(0.024)	0.331	(0.024)			0.339	(0.025)	0.340	(0.025)
*Diagnostics:*																		
*Measures of fit:*																		
AIC	154.260		128.480		127.093		674.497		676.484		676.949		685.361		686.825		687.719	
BIC	181.841		163.942		162.554		702.078		711.946		712.411		712.942		722.286		723.181	
*Tests for spatial error dependence:*																		
Lagrange multiplier stat.	35.919						1.790						2.580					
Lagrange multiplier *p*-value	0.000						0.181						0.108					
Moran’s I *z*-value	6.545						1.692						1.972					
Moran’s I *p*-value	0.000						0.091						0.049					
*Tests for spatial lag dependence:*																		
Lagrange multiplier stat.	42.511						1.657						1.733					
Lagrange multiplier *p*-value	0.000						0.198						0.188					
*Wald test of spatial terms:*																		
chi2			38.307		37.270				2.112		1.587				2.650		1.687	
Prob > chi2			0.000		0.000				0.146		0.208				0.104		0.194	

*Source:* own calculations based on county level data as described in *Source* notes for Figs. 1 and 3

*Notes:* Number of observations: 380. *p < 0.1, **p < 0.05, ***p < 0.01. Spatial error model – with spatially lagged errors, maximum likelihood estimator. Spatial lag model – with spatially lagged dependent variable, maximum likelihood estimator. The estimated variance inflation factor for conditioning variables varies between 1.66 and 3.78

Apart from the spatial nature of the three measures of deaths, our results presented in Tables 2 and 3 reflect also the correlations of the regional death statistics in 2020 with pre-pandemic levels of a number of local characteristics. The estimated coefficients in this case need to be treated with caution since the included indicators may correlate with regional characteristics, which due to lack of regional information cannot be controlled for. This in turn may bias the estimated coefficients on the variables included in the regressions. Having said that some of the estimated regularities seem intuitive. For example, COVID-19 deaths in Germany correlate significantly with the proportion of individuals in the oldest age group (85+), and overall excess deaths as well as the difference between excess and COVID-19 deaths are negatively related to the number of hospital beds per 1000 of population. Moreover, the difference between excess and COVID-19 deaths in Germany, i.e. the additional deaths over and above those officially recorded as COVID-19 deaths, are negatively correlated with population density and positively correlated with proportion of the population employed in agriculture. A possible interpretation of this finding could point towards increased levels of excess deaths in rural regions with lower levels of education. In Poland, such relationships are found for COVID-19 deaths, which seem to be higher in less densely populated regions with high levels of agricultural employment. The age group at highest risk of COVID-19 death in Poland seems to be those aged 70-84, and, surprisingly at first sight, COVID-19 deaths are higher in regions with more extensive hospital infrastructure. Possible explanations behind the latter could be factors related to healthcare infrastructure such as more extensive testing and lower likelihood of home (and thus untested) deaths. Importantly, we find few regularities in the regional pattern of excess deaths, while for the excess deaths over and above deaths due to COVID-19 we only find that these were lower in regions with higher agricultural employment.

As we can see in Tables 2 and 3 the spatial pattern of estimates in the spatial error and spatial lag models reflect the conclusions derived from the formal testing of spatial dependence discussed above. We find statistically significant coefficients on the respective spatial pattern in COVID-19 deaths in both Poland and Germany, while for overall excess deaths and for the differences between excess and COVID-19 deaths the spatial pattern is only identified in Germany. This confirms the key finding of the paper that excess deaths over and above the official COVID-19 deaths in Poland do not reflect the pattern of spatial dependence, which could be expected in a pandemic if its fatal consequences were direct consequences of infections or were indirect consequences of type 1, as per our definitions. The results suggest therefore that excessive deaths in Poland in 2020, over and above those officially registered as resulting from SARS-CoV-2 infections, have been dominated by indirect consequences of type 2. This may point towards important healthcare policy failures or policy neglect in the first year of the pandemic in Poland.

## Discussion

Using multivariate spatial autoregressive models we examine three measures of mortality in the first year of the COVID-19 pandemic in Germany and Poland: the officially recorded COVID-19 deaths, the total values of excessive deaths and the difference between the two. To explore the degree of regional variation between and within countries with respect to these outcomes, we link them to important regional characteristics such as population, health care and economic conditions.

In Germany all of the mortality measures show very strong spatial correlation, a feature of the data we would expect to observe in a pandemic. One could therefore argue that excess deaths in Germany – those identified as related to COVID-19 and those not identified as such – were directly linked to the spread of SARS-CoV-2 or were indirect consequences of type 1, as per our definitions. In Poland we also confirm spatial correlation of COVID-19 deaths. However, total excess deaths and the difference between excess deaths and COVID-19 deaths show no such pattern. Thus the deaths over and above the official COVID-19 deaths do not reflect the features we would expect to see for pandemic-related deaths, suggesting a significant proportion of deaths which were COVID-19’s indirect consequences of type 2.

The observed spatial regularities in Germany are consistent with the estimated coefficients on pre-pandemic regional characteristics. In particular, total excess deaths in Germany are negatively related to healthcare infrastructure, which supports the indirect influence of COVID-19 and its implications for the ability to treat other patients. We find little support for the expected role of county-level population density and for the industry structure of local employment. Both might be due to differentiated level of education and the related response in counties with lower density and an agricultural profile of the local economy. Moreover, the results may point towards the importance of more specific workplace characteristics rather than a simple sectoral division.

Our findings for Poland, where we do not observe the expected spatial pattern of total excess deaths and the excess deaths over and above the official COVID-19 deaths, could not be explained by the regional (pre-)conditions and require alternative explanations. This points to a high proportion of deaths resulting from indirect consequences of type 2, which in turn may relate to a number of policy deficits as well as individual reactions to the pandemic in Poland. First of all, during the pandemic individuals in Poland may have withdrawn from various healthcare interventions, principally as a result of fear of infection. Secondly, those with serious health conditions unrelated to the pandemic may have received insufficient care during the COVID-19 crisis in Poland, and, in consequence, died prematurely. This may have been a result of lower effectiveness of online medical consultations, excessive limitations to hospital admissions unjustified from the point of view of the spread of the virus, and/or worsened access to healthcare services resulting from lockdowns and mobility limitations. These deaths could also have resulted from reduced direct contact with other people (including family and friends as well as care personnel, etc.) and mental health deterioration as a consequence of (self)isolation. Our analysis does not allow us to differentiate between these hypotheses, but the aggregate excess deaths data suggest that the combination of these reasons came at a massive cost in terms of loss of lives. These excessive deaths still count as consequences of the COVID-19 pandemic in the sense that they would not have happened without it - we classify them as indirect consequences of type 2. They reflect a very particular type of failure in the areas of healthcare and public health in Poland. Given the heavy death toll as a consequence of this failure, specific reasons behind it deserve detailed examination.

Our analysis is mostly explorative in nature and not without limitations. The relationship between mortality and regional covariates described in the paper should be interpreted with caution, as the pre-pandemic regional indicators employed in the analysis might correlate with other unobserved regional characteristics, which may in turn bias the estimated coefficients. Moreover, the adopted approach to calculating excess deaths, while commonly used by established statistical institutions like the EUROSTAT [2] or in research studies [52–54], is in no way the only method. Alternative definitions often rely on models accounting for the population age composition and other potential determinants [1, 55–58]. However, as argued by Levitt and co-authors [59] and confirmed by others [1, 60], all such models require specific assumptions, which in turn translate into the nature of the predicted values – including their spatial dependence. From the point of view of the focus of this paper, the simple historical average thus, has the additional advantage that it is free from an a-priori assumption in this dimension. On top of that, it needs to be noted that local response to the pandemic also differed systematically between the two countries, and within them over space and time, since in Germany most public health and economic policies in the follow up of the outbreak of the pandemic remained in the hands of regional governments at the level of the *Länder*. Poland and Germany have followed different policies on the intensity of COVID-19 testing, test-and-trace policies, timing and intensity of social lockdowns, mask and distancing requirements, etc. [61, 62].^5^ The countries (and *Länder*) have also imposed different restrictions on access to medical staff and hospitals, and applied different policies concerning allocation of hospital beds for COVID-19 patients. Even if those developments could not be accounted for in our exploratory analysis, we present a first descriptive assessment and a new – spatial – angle to shed light on the (vast) regional variation in the consequences of the pandemic. The documented differences in spatial patterns of deaths provide strong motivation for further in-depth research aimed in particular at identification of causes behind the findings for Poland. We have shown here that country differences in the consequences of the ongoing pandemic can serve as a platform to set and test hypotheses about effectiveness of policy responses to future global health crises.

## Conclusions

Given the nature of the spread of SARS-CoV-2, strong regional patterns in the consequences of the COVID-19 pandemic were to be expected. These patterns could be mediated or strengthened by the regional variation of characteristics correlated with the likelihood of the spread of the virus and with the fatal consequences of infections. In this paper we conduct a detailed examination of the spatial patterns of mortality in the first year of the pandemic in Germany and Poland, which, among high income countries differ substantially in terms of the factors examined. The analysis shows that spatial patterns of deaths can provide important clues as to the reasons behind dramatic differences in the consequences of the COVID-19 pandemic across countries. These patterns in Germany, Poland and other countries ought to be further explored to inform design of public policy in response to global health crises.

## Supplementary Information

**Additional file 1.**

**Additional file 2.**

## Data Availability

The data used in this manuscript is publicly available, and was collected by the authors from different sources listed in details in the Data Statement, specifically for the purpose of this study. The analysis presented in the manuscript was prepared using Stata 15 software. Files with syntax enabling replication of the results are available at: 10.5281/zenodo.7529741.

## Footnotes

1 Administratively *Kreise* correspond to NUTS-3 level, while *powiaty* to LAU-1 (formerly NUTS-4) level in the NUTS classification.

2 According to the WHO guidelines published at a very early stage of the pandemic, COVID-19 death was registered in case of a confirmed diagnosis of COVID-19 (positive test result) or clinical or epidemiological diagnosis of the disease – suspected or probable [48]. These guidelines were followed in both Germany [49] and Poland [50]. This approach to counting COVID-19 deaths is most likely behind discrepancies with respect to the annual aggregated numbers of COVID deaths as reported by the national statistical offices in the death registers with the main cause of death division.

3 The alternative approach in which we set the classes in such a way that divides the legend into equal classes based on the variable values from the minimum to the maximum makes most of the map illustrations largely uninformative.

4 The European Health Consumer Index accounts for several aspects of healthcare quality such as survival rates, prevention efforts, range of services, accessibility of treatment or patient rights. For details see: [51].

5 As COVID-19 vaccines became available in the early 2021 their roll-out and vaccination rates have also been different. Given their timing, however, these developments do not affect the results in this paper.
