# Supplementary figures and images for: Deaths during the first year of the COVID-19 pandemic: insights from regional patterns in Germany and Poland
Source: BMC Public Health. 2023 Jan 26;23:177. doi: 10.1186/s12889-022-14909-9 (PMC9878483; doi:10.1186/s12889-022-14909-9)

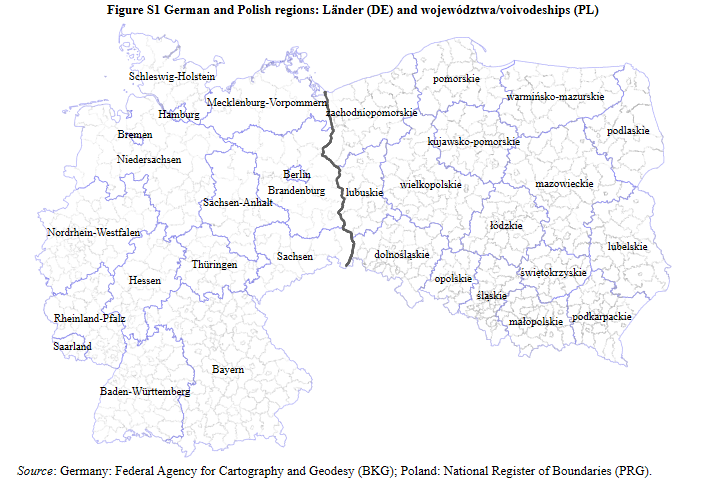

Supplement: Supplementary file 1 — Additional file 1. [file 12889_2022_14909_MOESM1_ESM.png]
