# Supplementary material for: Deaths during the first year of the COVID-19 pandemic: insights from regional patterns in Germany and Poland
Source: BMC Public Health. 2023 Jan 26;23:177. doi: 10.1186/s12889-022-14909-9 (PMC9878483; doi:10.1186/s12889-022-14909-9)
Supplement: Supplementary file 2 — Additional file 2. [file 12889_2022_14909_MOESM2_ESM.docx]

**Supplementary Material to the manuscript “Deaths during the first year of the COVID-19 pandemic: insights from regional patterns in Germany and Poland”**

**Table S1 Spatial autocorrelation of variables of interest calculated with the Moran’s I statistic (I) and the z-score using different spatial weight matrices**

|  | **Germany** | | | | | | **Poland** | | | | | |
| --- | --- | --- | --- | --- | --- | --- | --- | --- | --- | --- | --- | --- |
|  | **COVID deaths** | | **Excess mortality** | | **Difference** | | **COVID deaths** | | **Excess mortality** | | **Difference** | |
|  |  |  |  |  |  |  |  |  |  |  |  |  |
| Spatial weight matrix: | Moran’s I | z score | Moran’s I | z score | Moran’s I | z score | Moran’s I | z score | Moran’s I | z score | Moran’s I | z score |
|  |  |  |  |  |  |  |  |  |  |  |  |  |
| K-Nearest Neighbours: |  |  |  |  |  |  |  |  |  |  |  |  |
| First order | 0.517*** | 15.659 | 0.322*** | 9.724 | 0.114*** | 3.491 | 0.264*** | 7.849 | 0.060* | 1.831 | 0.062* | 1.894 |
| Second order | 0.418*** | 24.305 | 0.267*** | 15.501 | 0.104*** | 6.139 | 0.145*** | 8.380 | 0.019 | 1.238 | 0.015 | 0.991 |
| First*1, second*0.5 | 0.441*** | 24.298 | 0.281*** | 15.438 | 0.106*** | 5.920 | 0.168*** | 9.179 | 0.028 | 1.634 | 0.025 | 1.473 |
|  |  |  |  |  |  |  |  |  |  |  |  |  |
| Fixed distance with truncation: |  |  |  |  |  |  |  |  |  |  |  |  |
| - 70 km | 0.425*** | 23.506 | 0.254*** | 14.031 | 0.115*** | 6.418 | 0.150*** | 8.475 | 0.016 | 1.031 | 0.010 | 0.715 |
| - 80 km | 0.429*** | 27.001 | 0.274*** | 17.240 | 0.125*** | 7.940 | 0.131*** | 8.531 | 0.011 | 0.856 | -0.005 | -0.170 |
| - 90 km | 0.410*** | 29.185 | 0.265*** | 18.853 | 0.122*** | 8.771 | 0.119*** | 8.739 | 0.014 | 1.213 | -0.004 | -0.090 |
| - 100 km | 0.401*** | 31.481 | 0.247*** | 19.394 | 0.111*** | 8.761 | 0.103*** | 8.464 | 0.017 | 1.600 | 0.003 | 0.465 |
| - 110 km | 0.388*** | 33.650 | 0.238*** | 20.627 | 0.108*** | 9.463 | 0.092*** | 8.312 | 0.021** | 2.115 | 0.017* | 1.734 |
|  |  |  |  |  |  |  |  |  |  |  |  |  |
| Inverse distance with truncation: |  |  |  |  |  |  |  |  |  |  |  |  |
| - 70 km | 0.467*** | 21.539 | 0.269*** | 12.382 | 0.116*** | 5.395 | 0.191*** | 8.708 | 0.031 | 1.499 | 0.028 | 1.383 |
| - 80 km | 0.467*** | 23.970 | 0.282*** | 14.424 | 0.122*** | 6.293 | 0.176*** | 8.972 | 0.026 | 1.410 | 0.016 | 0.939 |
| - 90 km | 0.452*** | 25.583 | 0.275*** | 15.541 | 0.120*** | 6.855 | 0.164*** | 9.207 | 0.025 | 1.539 | 0.015 | 0.945 |
| - 100 km | 0.442*** | 27.000 | 0.262*** | 16.010 | 0.113*** | 6.969 | 0.152*** | 9.250 | 0.026* | 1.699 | 0.016 | 1.091 |
| - 110 km | 0.431*** | 28.405 | 0.256*** | 16.814 | 0.111*** | 7.390 | 0.142*** | 9.286 | 0.027* | 1.929 | 0.022 | 1.576 |
|  |  |  |  |  |  |  |  |  |  |  |  |  |

*Source:* own calculations based on county level data as described in *Source* notes for Figures 1 and 2.  *Notes: *p < 0.1, **p < 0.05, ***p < 0.01 for a two-tail test.*
